# Development and Comparison of Intramuscularly Injected Long-acting Testosterone Undecanoate Nano-/Microcrystal Suspensions with Three Different Particle Size

**DOI:** 10.22037/ijpr.2019.14408.12370

**Published:** 2021

**Authors:** Lihua Jia, Jiachao Xiao, Xi Yang, Jing Gao, Hui Zhang, Fanglin Yu, Aiping Zheng

**Affiliations:** a *Department of Pharmacy, Beijing Beiya Orthopedics Hospital, Beijing, China. *; b *State Key Laboratory of Toxicology and Medical Countermeasures, Beijing, China. *; c *Department of Pharmaceutics, Institute of Pharmacology and Toxicology, Beijing, China.*; 1 *L. J. and J. X. contributed equally to this work.*

**Keywords:** Testosterone undecanoate, Nanocrystal, Microcrystal, Particle size, Pharmacokinetics

## Abstract

The aim of this study was to develop and compare the pharmacokinetic property of testosterone undecanoate (TU) nano-/microcrystal suspension with three different particle sizes after intramuscular (i.m.) administration. TU nano-/microcrystal suspensions were prepared by high pressure homogenization method and the mean particle size was 0.30 ± 0.11 μm (A), 1.21 ± 0.37 μm (B), and 4.83 ± 0.60 μm (C), respectively. Scanning electron microscope (SEM) was employed to observe the morphology of nano-/microcrystal suspensions after operation. X-ray Powder diffraction (XRPD) confirmed the crystalline state of TU in nano-/microcrystal suspension. After storage at 4 °C and 25 °C under mechanical shaking for 2 months, physical and chemical stabilities of nano-/microcrystal suspensions were measured by particle size analyzer and high performance liquid chromatography. There was no obvious change in particle size distribution and content of TU. After i.m. administration of suspension C to rats, the concentration of TU in plasma lasted for nearly 12 days that was comparative with the commercial testosterone undecanoate injection. The results showed that microcrystal C with a larger particle size had long-acting effect comparing with other two suspensions. The muscle irritation test in rabbits showed that the local irritation of TU nano-/microcrystal suspensions was lower than that of commercial testosterone undecanoate injection. It can be concluded that appropriate particle size of nano-/microcrystal suspensions for i.m. administration of TU was important to achieve better therapeutic effect.

## Introduction

Late-onset hypogonadism (LOH) is a clinical and biological syndrome associated with advancing age and characterized by typical symptoms and a deficiency in serum testosterone levels. LOH may lead to significant detriment in the quality of life and adversely affect the function of multiple organ systems, including physical or mental frailty, body composition or bone mass, and prostate or cardiovascular systems. Testosterone undecanoate (TU) could significantly increase the serum testosterone level and improve the clinical symptoms of LOH patients without inducing serious adverse reactions. In clinical practice, TU soft capsules and TU injection are available for LOH Patients (1-4). However, most patients have poor compliance with TU soft capsules because they have to take the medicine twice a day for several days. Furthermore, the side effect to gastrointestinal tract caused by oleic acid contained in the soft capsule has taken much attention of scientists. TU injection contains benzyl benzoate and castor oil, which may produce allergic reactions, excess pain and pulmonary embolism. Therefore, the commercial products of TU for LOH therapy were less than ideal (5).

Sustained-release (SR) injections are designed to release a drug substance at a predetermined rate to maintain its effective plasma concentration for a specific period of time for months (6). Recently, nano-/microcrystal suspensions has emerged as a new, injectable SR formulation for facilitating the delivery of poorly water-soluble active pharmaceutical ingredients (APIs) and enhancing API bioavailability (7-9). Although only a few of commercial products based on this technology have been developed**, **it has gained increasing interest for drug delivery. One of the major advantages is that nano-/microcrystal injections provide significantly higher drug loading than traditional approach. Moreover, pain had been alleviated in the injection site as a result of less solvents or co-solvents contained in the formulation (10).

In this study, three TU nano-/microcrystal suspensions with different particle size for i.m. administration were prepared. They were characterized *in-vitro* by particle size and zeta potential analysis, scanning electron microscope (SEM) and powder X-ray diffraction (PXRD). Pharmacokinetics of TU nano-/microcrystal suspensions with different particle size was studied after intramuscular (i.m.) administration to rats compared with commercial testosterone undecanoate injection. Muscle irritation study in rabbits was employed to evaluate the safety of TU nano-/microcrystal preparations preliminarily.

## Experimental

*Materials*

Testosterone undecanoate was obtained from XIANJU PHARMA (Zhejiang，China). Poloxamer 188 was kindly supplied by BASF (Germany). Docusate sodium and sodium lauryl sulfate were supplied by Beijing Fenglijingqiu Trading Co., Ltd. Polysorbate 80 were purchased from Serva pharmaceutical factory. Polyethylene glycol 4000 was supplied by Janssen Pharmaceutica Inc. Commercial testosterone undecanoate injection was obtained from XIANJU PHARMA (Zhejiang，China). All chromatographic reagents were of HPLC grade, and they were purchased from Thermo Fisher Scientific. All of the other reagents were of the highest grade commercially available.

*Animals*

Healthy female Sprague-Dawley rats (200 ± 20 g) were provided by Experimental Animal Center of Beijing Institute of Pharmacology and Toxicology. Female New Zealand rabbits were supplied by Beijing Jinmuyang experimental animal breeding Co. Ltd. All the works related to animals were approved by the Animal Ethic Committee (ETHICS CODE Permit NO. SCXK-(Beijing) 2007-004). Each rat or rabbit used in this study was kept under standard conditions of temperature, humidity, and light. They were fasted but had free access to water over one night just before the experiment. 

*Preparation of TU nano-/microcrystal*

TU nano-/microcrystal suspensions were prepared by high pressure homogenization (AH-100D, ATS Engineering Inc.). Nano-/microcrystal suspensions with drug loading of 12.5% (w/v) were prepared by two steps. Firstly, the drug was dispersed in the aqueous solution containing different steric stabilizers (Hydroxypropyl cellulose (HPC), poloxamer 188, polyvinyl pyrrolidone-40(PVP-40), polyethylene glycol-4000(PEG-4000) or Tween 20) and charge stabilizers (docusate sodium or sodium lauryl sulfate) and prehomogenized at 200~300 bar for several minutes. Secondly, the mixtures were homogenized according to following details: 10 cycles at 500 bar, 10 cycles at 1000 bar and 10 cycles at 1300 bar to obtain uniform small particle size. Each cycle of homogenization was one minute. Particle size of TU nano-/microcrystal suspensions was analyzed after each cycle. The homogenization was performed at 4 °C to prevent drug from melting during the homogenization process. The nano-/microcrystal suspensions were collected into glass vials, labeled, stored at 4 °C, and used for subsequent tests (11-13).

*Particle size analysis and Zeta potential*

Particle size of TU nano-/microcrystal suspensions was analyzed by dynamic light scattering (DLS) using Zetasizer Nano ZS90 (Malvern Instruments, UK). Nano-/microcrystal suspensions were diluted with deionised water before analysis. The particle size distribution was expressed as mean diameter ± SD. Meanwhile, Zeta potential of diluted preparations was also measured. Each sample was measured in triplicate.

*Crystal morphology*

Morphology of three nano-/microcrystal suspensions was observed using SEM (Hitachi, Japan). Samples were ﬁxed on a brass stub by using double-sided tape directly and were gold coated by a sputter coater under 20 mA for 80s and voltage at 20 kV. 

*X-ray powder diffractometry (XRPD)*

Coarse TU powder, blank excipients mixtures, physical mixtures of TU powder and excipients, and TU nano-/microcrystal suspensions were studied by a D8 Advance X-Ray Diffractometer (Bruker, Germany) with a Cu source of radiation. Standard runs were carried out over a 2θ range of 10-30° and 0.04°/min as scanning rate.

*Saturation solubility research*

Saturation solubility value of TU nano-/microcrystals suspensions in pure water, phosphate buffered saline (PBS) (pH 7.4), 0.1% sodium dodecyl sulfate (SDS) (w/v, pH 7.4), and 0.2% SDS (pH 7.4) was assayed, respectively, compared with that of coarse TU powder suspensions. Nano-/microcrystal suspensions were added to each medium in a screw-capped vial. Then vial was shaken continuously at 37 °C for 24 h. Then they were centrifuged at 10000 rpm for 10 min and ﬁltered through a 0.22 μm membrane (14). The concentration of TU in filtrate which was saturation solubility of TU in that medium was analyzed by HPLC. The chromatographic separation was performed on a C_18_ column (4.6 × 250 mm, 5 μm; Phenomenex Corp., USA) at the wave length of 240 nm. The mobile phase was acetonitrile-isopropanol-water (45:45:10, v/v).

*Dissolution study*

The dissolution profile of the nano-/microcrystal suspensions was studied using ChP dissolution apparatus Type II (ZRS-8G; Tianda Tianfa Technology Co., China). The dissolution media was 900 mL distilled water containing 0.1% SDS, or 0.1M PBS (pH 7.4) which was kept at 37 ± 0.1 °C. The rotation speed of paddle was 50 rpm (15). At ﬁxed time intervals, 5 mL sample was withdrawn, ﬁltered through a 0.22 μm membrane filter, diluted with methanol and analyzed by HPLC for TU content. Analytic methods of HPLC were the same as saturation solubility research.

*Stability of TU nano-/microcrystal suspensions*

Nano-/microcrystal suspensions were sealed and stored at two different temperatures 4 °C and 25 °C under mechanical shaking for 2 months. Particle size distribution and crystallinity after storage were analyzed. 

*Animals and dosing*

The pharmacokinetic study was carried out using female Sprague-Dawley rats because testosterone was an endogenous substance. Twenty rats weighing 280 g~320 g were randomly divided into four groups which were administered with nano-/microcrystals suspensions with mean particle size of 0.30 ± 0.11 μm (A), 1.21 ± 0.37 μm (B), and 4.83 ± 0.60 μm (C) as well as commercial testosterone undecanoate injection (D), respectively. The animals were fasted overnight (with free access to water) before the experiments. The rats were administered with normal saline (NS) containing nano-/microcrystals suspensions or commercial testosterone undecanoate injection by i.m. injection (25mg/kg). Blood samples were collected from orbital veins of rats at fix times and centrifuged at 3500 rpm for 10 min. Then plasma sample was separated and frozen at -20 °C until analysis.

*Determination of testosterone concentrations in plasma*

Testosterone undecanoate was metabolized to testosterone after entering blood circulation because of the esterase (16). The concentration of testosterone in plasma was analyzed by LC-MS/MS (Agilent Technologies, 6460 Triple Quad LC-MS), using L-phencynonate hydrochloride as the internal standard. After 100μl of plasma sample was mixed with 100μL internal standard solution (2 ng/mL), 200μL of methanol was added to the mixture in order to precipitate the protein. The plasma samples were fully mixed by the vortex mixer, and then were centrifuged at 14000 rpm for 10 min. The supernatant layer was transferred into a vial, and 5μL was injected into LC-MS/MS system.

The chromatographic separation was performed on a C_18_ column (100 x 2.1 mm, 5μm; Thermo Corp., USA) at a column temperature of 40 °C. The mobile phase consisted of 0.05% formic acid and 5mmol ammonium formate aqueous solution (A) and methanol (B). The autosampler temperature was set at 4 °C (8). Testosterone and IS were analyzed by multiple reaction monitoring (MRM) of the transitions of m/z 289.1→97.1 and m/z 289.1→109.1 for testosterone, m/z 385.2 →267.3 for IS, respectively (17).

*Pharmacokinetic data analysis*

The data were calculated and analyzed by DAS 2.0 software, including area under the plasma concentration time curve (AUC_(0-∞)_) and mean residence time (MRT_(0-∞)_). Maximum plasma concentration of testosterone (C_max_) and time to reach the peak plasma concentration (T_max_) were obtained directly from plasma concentration versus time profiles.

*Muscle irritation study in rabbits*

The method applied in this study was under the guidelines of CFDA ^(^^18^^)^. Female New Zealand rabbits (2.5-3.0 kg) were used in this study to compare muscle irritation of several preparations after i.m. injection. NS was added to different preparation in order to adjust the osmotic pressure values to 280-320 mOsmol/ kg before injection. Seven rabbits were divided into seven groups at random, injected with nano-/microcrystal suspensions with three different particle size, commercial testosterone undecanoate injection, excipients preparation without TU, acetic acid solution of 0.425% (W/V) as positive control and NS as blank control, respectively. Rabbit was injected with 2mL on the right side of the quadriceps femoris. The other side was injected with 2mL of NS. After 48 h, seven rabbits were sacrificed. The quadriceps femoris on the injection site of the rabbits was removed and prepared for histopathologic examination.

*Statistical analysis*

The values were expressed as mean ± SD (standard deviation). Differences between the groups were assessed using the paired, two sided Student’s t-test. *p < 0.05 was considered as signiﬁcant.

## Result and Discussion

*Preparation of testosterone undecanoate nano-/microcrystal suspensions*

In the preparation process，there were several factors that affect the properties of nano-/microcrystal suspensions, including formulation composition, homogenization pressure, and cycles. Physically stable nano-/microcrystal suspensions were obtained when the stabilizer could be capable of wetting the surface of the drug crystals and providing a steric or ionic barrier. Selection of stabilizers was critical to obtain drug particle within nanometer range. Nano-/microcrystal suspensions tend to be unstable because of agglomeration, precipitation or crystal growth which leads to the decrease of total system energy (19-20). Besides, solubility and molecular weight of the drug, surface energy and interactions between specific functional groups affect the steady state of nano-/microcrystal suspensions (21-22). Meanwhile, high concentration of stabilizer may be required because of high drug loading demand. Considering the maximum dosage of surfactant used in muscle injections published by FDA, testosterone undecanoate nano-/microcrystal suspensions were prepared with PEG 4000 (5%, w/w) and Tween 20 (2%, w/w) as the steric stabilizer. Under the low homogenization pressure of 500 bar, the sizes of nano-/microcrystal suspensions were decreased to 4.83 ± 0.60 μm after 10 cycles. As the homogenization pressure was enhanced to 1000 bar, the sizes of nano-/microcrystal suspensions was decreased to 1.21 ± 0.37 μm after 10 cycles. As the homogenization pressure was enhanced to 1300 bar, the sizes of nano-/microcrystal suspensions was decreased to 0.30 ± 0.11 μm suspension when the cycle times was increased from 1 to 8 but displayed no significant change as the cycle times was increased up to 10 (11-13). At last, 30 mL of nano-/microcrystal suspensions for each particle size was collected. 

*Morphology, particle size and zeta potential*

**Figure 1:**
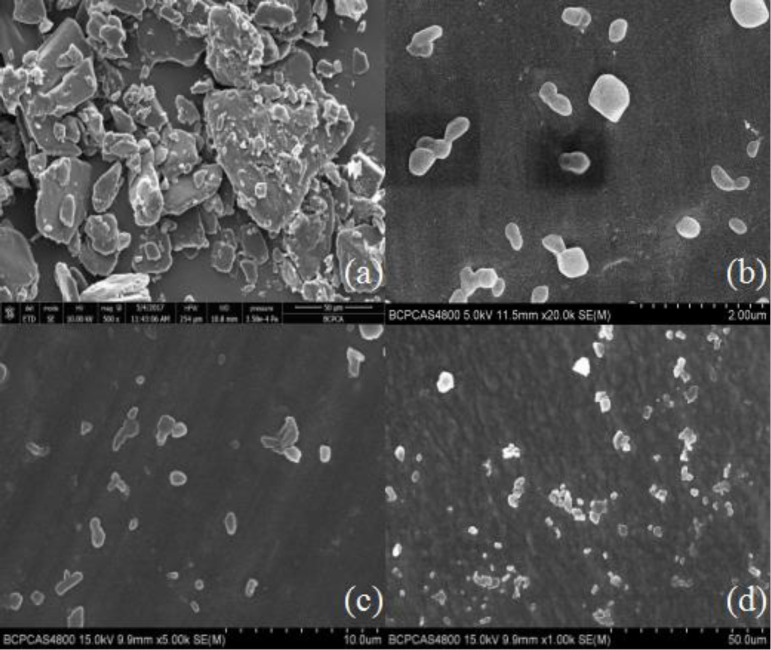
SEM of (A) suspension of drug powder, (B) A suspension, (C) B suspension, (D) C suspension

Nano-/microcrystal suspensions with drug loading of 12.5% (w/v) of testosterone undecanoate were achieved. To characterize the morphology of the coarse TU and TU nano-/microcrystal suspension, SEM was performed. As Shown in Figure 1, TU raw material presented prismatic and tabular shape, and TU nano-/microcrystal suspension had ordered granular shape. Change in the morphology might be attributed to high pressure homogenization process of the coarse drug. During the homogenization process, collision and shear forces broke the drug down to nano-/micrometer range and changed the morphology of TU raw material (23).

**Figure 2:**
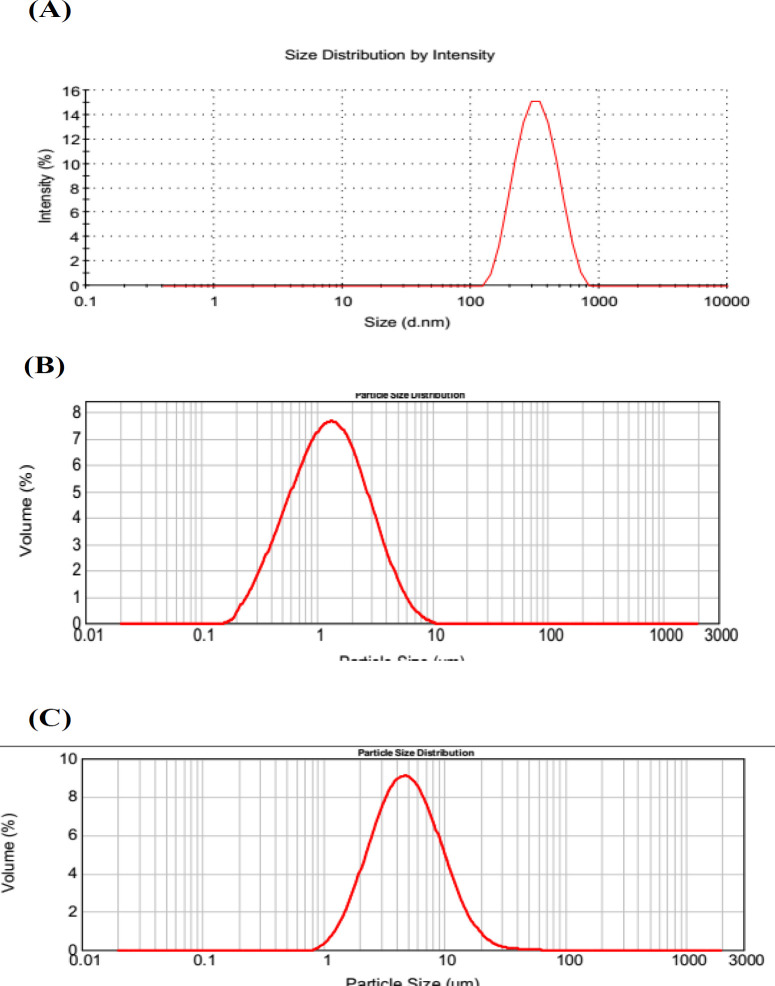
Particle size distribution of (A) A suspension, (B) B suspension, (C) C suspension

Particle size distribution of three nano-/microcrystal suspensions analyzed by DLS was shown in Figure 2. Zeta Potential of diluted nano-/microcrystal suspensions with different particle size was 32.6 ± 1.49 mV, 30.2 ± 1.27 mV, and 31.2 ± 1.58 mV, respectively. As a reﬂection of barrier that can prevent nanosized particles from aggregating, zeta potential had a signiﬁcant effect on the stability of nanosuspensions. Generally, zeta potential of a physically stable system should be at least 20mV for sterically stabilized nanosuspensions, and also 30 mV for electrostatically stabilized nanosuspensions(24). In this study, TU nanosuspension was protected by electrostatical effect with zeta potential of 32.6 ± 1.49mV which is enough to keep the stability.

*XRPD*

**Figure 3:**
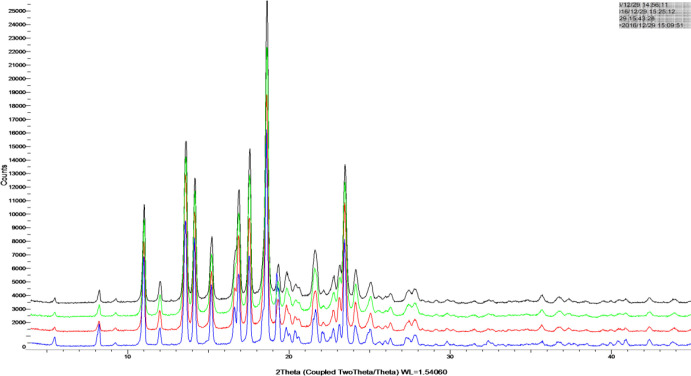
XRPD diagrams of TU drug powder, A suspension , B suspension , C suspension

The XRPD diagrams of TU drug powder and nano-/microcrystal suspensions were shown in Figure 3 Diffraction peaks were observed for TU at 2θ of 19.26° and 23.40°. The characteristic diffraction peaks for nano-/microcrystal suspensions with three different particle size could be still identiﬁed, conﬁrming the crystalline state TU in nano-/microcrystal suspensions. The XRPD diagrams demonstrated that TU did not undergo a polymorphism transition.

*Saturation solubility and in-vitro dissolution research*

**Figure 4:**
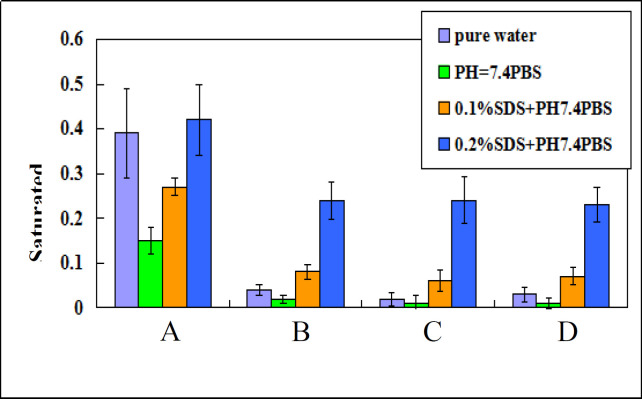
Saturated solubility of (A) A suspension, (B) B suspension, (C) C suspension, (D) TU drug powder in different mediums

Based on Ostwald-Freundlich equation, saturation solubility of water-insoluble drug increases when particle size changes from micrometer range to nanometer range. Saturation solubility of nano-/microcrystal suspensions with three different particle size at 37 °C in water, PBS (PH 7.4), 0.1%SDS and 0.2%SDS were shown in Figure 4. Saturation solubility of nanocrystal suspension in these four mediums was higher than that of drug powder or microcrystal suspensions. 

**Figure 5:**
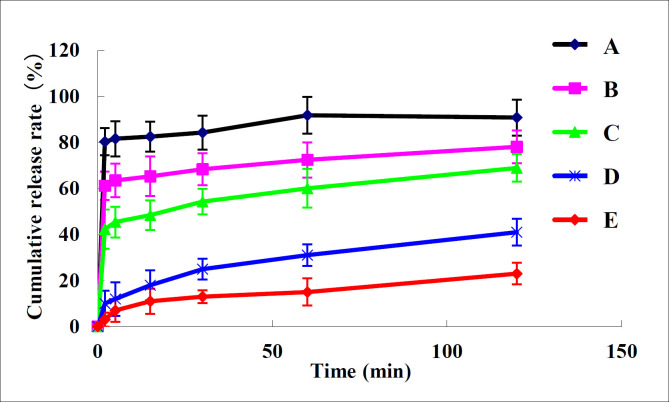
Dissolution proﬁles of (A) A suspension, (B) B suspension, (C) C suspension, (D) TU drug powder, (E) commercial testosterone undecanoate injection in different mediums

Dissolution behavior of nano-/microcrystal suspensions compared with TU drug powder and commercial testosterone undecanoate injection was shown in Figure 5. It can be concluded that dissolution of nano-/microcrystal suspensions was much higher than that of drug powder and commercial testosterone undecanoate injection. Dissolution rate of TU increases when particle size reduces to nanometer range because smaller particle size indicated larger surface area (25).

*Stability of nano-/microcrystal suspensions*

 Although some aggregates appeared from visual observation during the period of storage, nano-/microcrystal suspensions could be easily redispersed by gentle shaking (26). Mean particle size of three nano-/microcrystal suspensions presented no signiﬁcant change stored at 25 °C, 4 °C and mechanical shaking condition within 2 months (*p *> 0.05). The TU content (%) maintained steadily at a range of 98.0%–102.0%. Therefore, three nano-/microcrystal suspensions showed good physical and chemical stability.

*Pharmacokinetics in rats*

**Figure 6:**
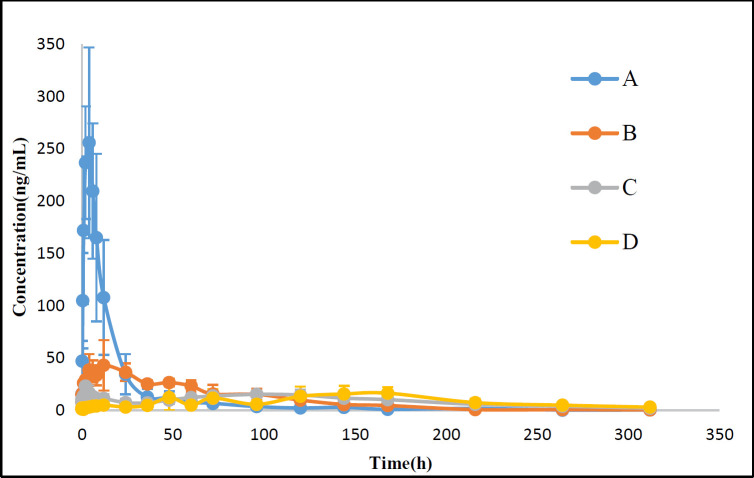
Plasma concentration-time curves for testosterone following i.m. administration of TU nano-/microcrystal suspensions (25 mg/kg) in female SD rats (A) A suspension, (B) B suspension, (C) C suspension (D) Commercial testosterone undecanoate injection

Because of the existence of endogenous testosterone in the plasma of male SD rats, female SD rats were used to evaluate the pharmacokinetics after administration with TU commercial testosterone undecanoate injection or suspensions with different particle size. The pharmacokinetic analysis was carried out by using DAS 2.1.1. The plasma concentration-time curves and the pharmacokinetic parameters for TU were shown in Figure 6 and Table 1. 

Following an injection, C_max_ of TU for nano-/microcrystal suspensions with different particle size and commercial testosterone undecanoate injection was 260.4 ± 88.9, 51.6 ± 15.4, 29.5 ± 11.3, and 18.5 ± 8.0 ng/mL, respectively. T_max_ for nano-/microcrystal suspensions increased when particle size increased. C_max_ and AUC_(0-∞)_ were signiﬁcantly increased with particle size reduced to nanometer range. Nanocrystal suspension with a small particle size had a higher C_max_ and AUC_(0-∞)_ compared with microcrystal suspension. MRT_(0-∞)_ of microcrystal suspensions with particle size of 4.8μm and commercial testosterone undecanoate injection was 137.8 ± 39.6 h and 184.3 ± 55.1 h, respectively, which indicated slower release from the injection site than nanocrystal suspension. These data demonstrated that microcrystal suspension with particle size of 4.83 ± 0.60 μm was long-acting formulation which was similar to the commercial testosterone undecanoate injection. TU was absorbed slowly after i.m. administration with microcrystal suspension probably because the relative small particles dissolved first, and then the large particles dissolved slowly until several days later (27-28). These results indicated that TU nano-/microcrystal suspensions have promising future as a long-acting drug delivery system of poor water soluble drug TU. 

*Muscle irritation study in rabbits*

**Figure 7:**
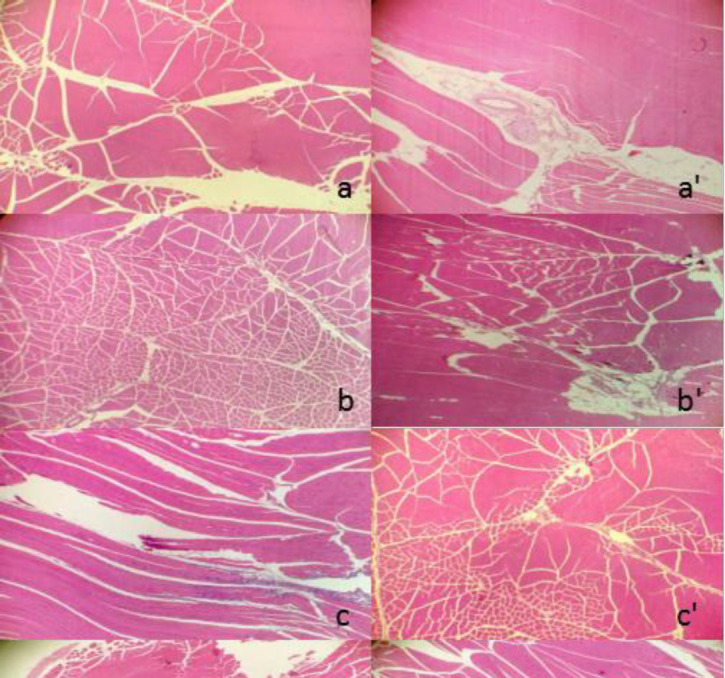
The transverse pathological section of the left side followed by injection of A suspension (A), B suspension (B), C suspension (C), commercial testosterone undecanoate injection (D), blank excipients solution (e), acetic acid solution of 0.425% (W/V) (f) and physiological saline group (g), respectively. The right side was corresponding longitudinal pathological section

No clinical signs and weight changes were observed over the period after i.m. administration with nano-/microcrystal suspensions or commercial testosterone undecanoate injection. Two days later, no obvious irritant reaction of those four preparations in the injection site was observed by macroscopic observation. Under light microscope, it showed that irritant reaction of commercial testosterone undecanoate injection group in muscle was different from that of nano-/microcrystal suspensions group and physiological saline group. Nano-/microcrystal suspension groups and blank excipients solution group exhibited the same irritant reaction of exudation of inflammatory cells. However, commercial testosterone undecanoate injection group induced hyperplasia of connective fibers, vascular degeneration, and slight inflammatory infiltration. All the results were shown in Figure 7. There was a report indicating that long term use of commercial testosterone undecanoate injection may cause induration on injective site related to large amount of oil which also induce pain feeling. 

**Table 1:** Pharmacokinetic parameters following i.m. administration of TU nano-/microcrystal suspensions (25 mg/kg) in rats

**Parameters**	**A suspension**	**B suspension**	**C suspension**	**Commercial testosterone undecanoate injection**
C_max _(ng/mL)	260.4 ± 88.9	51.6 ± 15.4	29.5 ± 11.3	18.5 ± 8.0
T_max _(h)	3.2 ± 1.7	7.0 ± 5.0	96.0 ± 49.8	108.0 ± 57.1
MRT_(0-∞)_ (h)	44.0 ± 11.9	69.2 ± 21.1	137.8 ± 39.6	184.3 ± 55.1
AUC_(0-∞) _(ng/mL/h)	4291.5 ± 1591.8	3322.9 ± 661.7	3824.1 ± 770.5	3196.0 ± 1673.2

*Data were expressed as mean ± SD, n = 5.

## Conclusion

Drug nanocrystals are a highly feasible option for enhancing drug release of poorly soluble drugs. In this study, injectable nano-/microcrystal suspensions containing 12.5% (w/v) TU with three different particle size were prepared using high-pressure homogenization method. Crystalline state of TU was not altered during particle size reduction. The nano-/microcrystal suspensions could be stored for 2 months in an aqueous state. There was no change in particle size and content of TU drug within 2 months that indicated good physical and chemical stability of the nano-/microcrystal suspensions. Muscle irritation study in rabbits showed that irritant reaction of nano-/microcrystal suspensions was significantly less than the commercial testosterone undecanoate injection. *In-vivo* pharmacokinetic study of TU nano-/microcrystal suspensions demonstrated that the suspensions we prepared may be a promising long-term late-onset hypogonadism medication, compared with nanocrystal suspension with a smaller particle size.

## References

[1] Tong SF, Ng CJ, Lee CB, Lee VKM, Khoo EM, Lee EG, Tan HM (2012). Effect of long-acting testosterone undecanoate treatment on quality of life in men with testosterone deficiency syndrome: a double blind randomized controlled trial. Asian. J. Androl..

[2] Haider A, Yassin A, Rosano GM (2016). Men with testosterone deficiency and a history of cardiovascular diseases benefit from long-term testosterone therapy: observational, real-life data from a registry study. Vasc. Health Risk Manag.

[3] Jiang T, Zheng L, Jiang H (2013). Long-term testosterone supplementation is useful for ED with testosterone deficiency. Asian J. Andrology.

[4] Almehmadi Y, Yassin AA, Saad F (2016). Testosterone replacement therapy improves the health-related quality of life of men diagnosed with late-onset hypogonadism. Arab. J. Urol..

[5] Ahmed A, Greenwood N (1973). Lymphadenopathy following repeated oil-based injections. J. Pathol..

[6] Salem HF (2010). Sustained-release progesterone nanosuspension following intramuscular injection in ovariectomized rats. Int. J. Nanomed..

[7] Junghanns JU, Müller RH (2008). Nanocrystal technology, drug delivery and clinical applications. AAPS PharmSciTech. Int J Nanomedicine..

[8] Sun B, Yeo Y (2012). Nanocrystals for the parenteral delivery of poorly water-soluble drugs. AAPS PharmSciTech. Curr Opin Solid State Mater Sci..

[9] Elaine ML, Liversidge GG, Cooper ER (2003). Nanosizing: a formulation approach for poorly-water-soluble compounds. Eur. J. Pharm. Sci..

[10] Klooster G, Hoeben E, Borghys H, Looszova A, Bouche MP, van Velsen F, Lieven Baert L (2010). Pharmacokinetics and disposition of rilpivirine(TMC278) nanosuspension as a long-acting injectable antiretroviral formulation. Antimicrob. Agents CH..

[11] Salazar J, Müller RH, Möschwitzer JP (2014). Combinative particle size reduction technologies for the production of drug nanocrystals. J. Pharm. (Cairo).

[12] Sattar A, Chen D, Jiang L, Pan Y, Tao Y, Huang L, Liu ZH, Xie S, Yuan Z (2017). Preparation, characterization and pharmacokinetics of cyadox nanosuspension. Sci. Rep..

[13] Fernandes AR, Ferreira NR, Souto EB (2017). Ibuprofen nanocrystals developed by 22 factorial design experiment: A new approach for poorly water-soluble drugs. Saudi Pharm. J..

[14] Peltonen L, Strachan C (2015). Understanding Critical Quality Attributes for Nanocrystals from Preparation to Delivery. Molecules.

[15] Ravouru N, Venna RSA Penjuri SCB, Damineni S, Kotakadi VS, Poreddy SR (2018). Fabrication and Characterization of Gliclazide Nanocrystals. Adv. Pharm. Bull..

[16] Forsdahl G, Vatne HK, Geisendorfer T, Gmeiner G (2013). Screening of testosterone esters in human plasma. Drug Test Anal..

[17] Xu W, Li H, Qing G, Shen L, Cheng L (2017). A rapid and simple liquid chromatography-tandem mass spectrometry method for the measurement of testosterone, and rostenedione, and dehydroepiandrosterone in human serum. J. Clin. Lab. Anal..

[18] (2005). CFDA: Technical guidelines for the study of chemical, irritant, allergic, and hemolytic agents.

[19] Lennart L, Pia S, Urban S, Rasmusson M, Zackrisson A, Olsson U (2006). Amorphous drug nano-/microcrystal. Inhibition of Ostwald ripening. Langmuir.

[20] Lennart L, Pia S, Urban S, Westergren J, Olsson U (2007). Amorphous drug nano-/microcrystal Particle dissolution and crystal growth. Langmuir.

[21] Tuomela A, Hirvonen J, Peltonen L (2016). Stabilizing Agents for Drug Nanocrystals: Effect on Bioavailability. Pharmaceutics..

[22] Gigliobianco MR, Casadidio C, Censi R, Martino PD (2018). Nanocrystals of Poorly Soluble Drugs: Drug Bioavailability and Physicochemical Stability. Pharmaceutics..

[23] Yadav SK, Mishra S, Mishra B (2012). Eudragit-based nanosuspension of poorly water-soluble drug: formulation and in-vitro–in-vivo evaluation. AAPS PharmSciTech..

[24] Ahmed TA (2016). Preparation of finasteride capsules-loaded drug nanoparticles: formulation, optimization, in-vitro, and pharmacokinetic evaluation. Int J Nanomedicine..

[25] Wei X, Wei S, Ke GL, Zhang FZ, Wai SC, Juan L, Yuan JH, Hua Y, Wang YT (2018). Dual-functional Brij-S20-modified nanocrystal formulation enhances the intestinal transport and oral bioavailability of berberine. Int. J. Nanomedicine..

[26] Badawi AA, El-Nabarawi MA, El-Setouhy DH, Alsammit SA (2011). Formulation and stability testing of itraconazole crystalline nanoparticles. AAPS PharmSciTech..

[27] Wong J, Brugger A, Khare A, Chaubal M, Papadopoulos P, Rabinow B, Kipp J, Ning J (2008). Suspensions for intravenous (IV) injection: A review of development, preclinical and clinical aspects development, preclinical and clinical aspects. Adv. Drug Deliv. Rev..

[28] Leng D, Chen H, Li G, Guo M, Zhu Zh, Xu L, Wang Y (2014). Development and comparison of intramuscularly long-acting paliperidone palmitate nanosuspensions with different particle size. Int. J. Pharm..

